# Linking transformational leadership, patient safety culture and work engagement in home care services

**DOI:** 10.1002/nop2.386

**Published:** 2019-10-08

**Authors:** Eline Ree, Siri Wiig

**Affiliations:** ^1^ SHARE – Centre for Resilience in Healthcare Faculty of Health Sciences University of Stavanger Stavanger Norway

**Keywords:** job demands, job resources, patient safety culture, transformational leadership, work engagement

## Abstract

**Aim:**

To assess the relationship between transformational leadership, job demands, job resources, patient safety culture and work engagement in home care services.

**Design:**

Cross‐sectional survey.

**Methods:**

Healthcare professionals in Norwegian home care services participated in the study (*N* = 139). Multiple regression analyses with patient safety culture and work engagement as outcomes and transformational leadership, job demands, job resources as predictors were conducted.

**Results:**

The transformational leadership model explained 35.7% of the variance in patient safety culture. Adding job demands and resources and work engagement to the model increased the explained variance to 53.5%. The job resource “skill utilization” was the strongest predictor of work engagement. The full model with all predictor variables explained 28.2% of work engagement.

**Conclusion:**

Transformational leadership has a significant impact on patient safety culture and work engagement in home care services. Employees' perceptions of job demands, available resources and engagement also affect patient safety culture.

## 1 INTRODUCTION

In this study, we explore whether transformational leadership, job demands and job resources influence patient safety culture and work engagement in Norwegian home care services. *Patient safety culture* can be defined as “the product of individual and group values, attitudes, perceptions, competencies and patterns of behaviour that determine the commitment to and the style and proficiency of, an organization's health and safety management” (ACSNI, 1993). Research has shown that a poor patient safety culture is associated with injuries to both nurses and patients (Taylor et al., 2012). A good safety culture is associated with patients' safety‐related outcomes, such as increased patient satisfaction (Hofmann & Mark, 2006) and fewer adverse events (Wang et al., 2014). To increase knowledge on how to improve patient safety culture, more research on the factors that affect it is needed.

In a comprehensive review of the literature on safety culture in the U.S. hospital setting, it is suggested that a “culture of safety begins with leadership” (Sammer, Lykens, Singh, Mains, & Lackan, 2010). The authors argue that leaders have a key role in designing, encouraging and developing a safety culture. The role of patient safety culture and leadership has also been pinpointed as key challenges in public inquiries into system failures that were allowed to develop over years and caused serious injury of death of numerous patients, as in the Mid staffordshire (Francis, 2013) and Bristol cases (Kennedy, 2001). The aftermath of these healthcare disasters has focused on managers' responsibility in building cultures for safety and learning.

Several studies emphasize the role of leaders in building a safety culture (Merrill, 2015; Wagner et al., 2018; Wong, Cummings, & Ducharme, 2013). McFadden, Henagan, and Gowen (2009) found that transformational leadership was directly related to patient safety culture and indirectly related to patient safety outcomes through culture and patient safety initiatives, such as education and training of employees and system redesign. Transformational leadership occurs “when leaders broaden and elevate the interests of their employees, when they generate awareness and acceptance of the purposes and mission of the group and when they stir their employees to look beyond their own self‐interest for the good of the group” (Bass, 1991, p. 21). In a meta‐analytic review, Clarke (2013) suggested that transformational leadership was associated with encouraging employee participation in safety. Furthermore, a systematic review of the relationship between nursing leadership and patient outcomes concluded that transformational leadership is positively related to improved patient outcomes, such as increased patient satisfaction and fewer adverse events and complications (Wong et al., 2013).

The relationship between transformational leadership and patient safety might partly be a result of managers' role in creating a sound work environment that balances job demands and resources (Boamah, Laschinger, Wong, & Clarke, 2018), as well as their influence on employees' work engagement (Salanova, Lorente, Chambel, & Martínez, 2011). According to Salanova et al. (2011), there is a direct link between transformational leadership and employees' work engagement. However, based on the job demand and job resources model arguing that engagement is largely a result of job resources and manageable job demands (Bakker & Demerouti, 2007), the link might also be indirect through working conditions. A study of acute care nurses in Ontario, Canada, found that transformational leadership behaviours had a positive influence on workplace conditions, which were then related to fewer adverse patient outcomes and greater job satisfaction among nurses (Boamah et al., 2018).

Several studies argue for the importance of work environment for patient safety (Nahrgang, Morgeson, & Hofmann, 2011; Olds, Aiken, Cimiotti, & Lake, 2017; Sturm et al., 2019). Similarly, employee engagement is found to be associated with patient safety culture (Biddison, Paine, Murakami, Herzke, & Weaver, 2016). A review by Mossburg and Dennison (2018) found that most studies reported a moderately strong association between engagement and patient safety culture. The review concluded, however, that the engagement/safety literature is immature and there is a need for more research on this topic.

National health policy documents and patient safety campaigns and programs have shown a strong focus on building structures and a culture for patient safety and that leaders are playing a key role in its success. In Norway, for example, the national campaign “In Safe Hands” was launched in 2011 by the Norwegian Ministry of Health and Care Services, to improve patient safety and reduce patient harm by highlighting leadership and the safety culture (Skjellanger et al., 2014). A new national regulation in Norway emphasizes managers' responsibility for quality and patient safety work (Ministry of Health & Care Services, 2016).

## 2 BACKGROUND

The research on leadership in relation to patient safety in the home care context is scarce. Patient safety research in home care services is limited, as most of the literature on safety culture centres on hospitals and acute care settings (Gartshore, Waring, & Timmons, 2017) in addition to some recent studies from Norwegian nursing homes (Cappelen, Aase, Storm, Hetland, & Harris, 2016; Cappelen, Harris, & Aase, 2018). One study has explored perceptions of the patient safety culture in Norwegian home care services (Olsen & Bjerkan, 2017). Furthermore, to the best of our knowledge, no studies have explored the impact of transformational leadership on patient safety culture and work engagement in home care services and whether job demands, and resources help to explain these outcomes. The aims of this study are therefore to explore (a) the degree to which transformational leadership, job demands and resources and work engagement can explain patient safety culture in home care services; and (b) the degree to which transformational leadership and job demands and resources, can explain work engagement among home healthcare personnel. Based on previous research and theory, the following hypotheses are postulated:

Hypothesis 1: Transformational leadership, job resources and work engagement are positively related to patient safety culture, while job demand is negatively related to patient safety culture.

Hypothesis 2: Transformational leadership is a stronger predictor for patient safety culture than job demands, job resources and work engagement.

Hypothesis 3: Transformational leadership and job resources are positively related to work engagement, while job demand is negatively related to work engagement.

Hypothesis 4: Transformational leadership is a stronger predictor for work engagement than job demands and job resources.

## 3 METHODS

This study used a cross‐sectional survey design.

### 3.1 Setting, sample and data collection

The questionnaire was distributed to 206 participants, with 139 responding (response rate of 67.5%). Co‐researchers from the Development Centres of Nursing Home and Home Care Services helped with the recruitment by initiating contact with the unit managers of the included home care units (Wiig et al., 2018). A purposeful sampling strategy was used, strategically selecting four units from four municipalities in south‐western Norway that varied in size, unit size and location (urban and rural) (Table 1). Inclusion criteria were that the employees had to be employed in at least a 30% position and capable of writing in the Norwegian language. Questionnaires were distributed through e‐mail to all participants in April 2018.

**Table 1:** Study setting

	Unit	Municipality
HCS A	250–300 users ca. 30 min to hospital	Large district 15–20,000 inhabitants
HCS B	200–250 users ca. 20 min to hospital	City 70–75,000 inhabitants
HCS C	200–250 users ca. 1 hr to hospital	Small district <5,000 inhabitants
HCS D	50–100 users ca. 20 min to hospital	Medium‐sized district 5,000–10,000 inhabitants

In Norway, home care services and nursing homes are organized and provided by municipalities, implying that the management roles and responsibilities are similar in both settings. Moreover, this implies that all healthcare staff in both nursing homes and home care are municipal employees. Municipalities are responsible for primary care services in Norway such as general practitioners, emergency rooms, nursing homes and home care services. The organizational structure of the units differed somewhat because of variations in geography, size and local circumstances. In the smallest municipality (HCS C), employees often shared responsibility across departments and could take shifts in both nursing homes and home care services as needed. However, they responded to the home care survey if they worked most of their position in the home care services. All home care services provided home nursing and practical assistance to people in need of such services. All municipalities were organized under a director of health and welfare services at the municipal level who was responsible for all healthcare services delivered in the municipality. In our study, each unit had a unit manager with personnel responsibility for the whole organization. Three of the units (HSC B–D) also had one department manager, while the largest unit (HCS A) had three department managers, one for each of the three departments in the unit.

### 3.2 Instruments

#### 3.2.1 Patient safety culture

Patient safety culture was measured by the Norwegian version of the Nursing Home Survey on Patient Safety Culture (NHSOPSC), developed and validated by Cappelen et al. (2016). The instrument consists of 41 items that measure health personnel's perceptions of patient safety culture, rated on 5‐point Likert scales. In this study, some of the items in the Norwegian nursing home version by Cappelen et al. (2016) were slightly adjusted to fit the home care setting. The only adjustments made were the change in some words, for instance, replacing “nursing home” with “unit” or “home care.” In this study, all 41 items were computed as a mean score and used as an outcome measuring the total patient safety culture score. The Cronbach's alpha value of this total score was .88. Previous studies have demonstrated an acceptable fit of a 10‐factor model of the scale in Norwegian settings (Cappelen et al., 2016, 2018). The 10 factors are “teamwork,” “staffing,” “compliance with procedures,” “training and skills,” “nonpunitive responses to mistakes,” “handoffs,” “feedback and communication about incidents,” “communication and openness,” “supervisor expectations and actions promoting patient safety” and “organizational learning” (Cappelen et al., 2016).

#### 3.2.2 Transformational leadership

Transformational leadership was measured using the Global Transformational Leadership Scale (GTL) (Carless, Wearing, & Mann, 2000). Staff were asked to rate the leadership style of their nearest leader on seven items rated on 5‐point Likert scales ranging from “never” to “very often.” Examples of items are as follows: “Encourages thinking about problems in new ways and questions assumptions; and “Treats staff as individuals, supports and encourages their development.” Carless et al. (2000) found GTL to have high degree of convergent validity with the more extensive and established Multifactor Leadership Questionnaire (MLQ) (Avolio, Bass, & Jung, 1995). In this study, Cronbach's alpha was .93.

#### 3.2.3 Job demands and job resources

Job demands and job resources were measured with the Short Inventory to Monitor Psychosocial Hazards (SIMPH) (Notelaers, De Witte, Van Veldhoven, & Vermunt, 2007), which is based on the Job Demands–Resources model by Demerouti, Bakker, Nachreiner, and Schaufeli (2001). The scale consists of 24 items rated on 4‐point Likert scales (1 = never to 4 = always). Job demands consist of three dimensions: (a) pace of work (4 items, e.g. “Do you work under time constraints?”); (b) mental workload (3 items, e.g. “Do you have to focus your attention on several things simultaneously?”); and (c) emotional workload (5 items, e.g. “Is your work heavy from an emotional viewpoint?”). Cronbach's alpha values were .91 (pace of work); .81 (mental workload); and .77 (emotional workload). Job resources consist of three dimensions: (a) skill utilization (4 items, e.g. “Do you feel that you achieve something meaningful in your job?”); (b) autonomy (four items, e.g. “Do you have an influence on the pace of work?”); and (c) participation (4 items, e.g. “Can you participate in decisions affecting areas related to your work?”). Cronbach's alpha values were .77 (skill utilization), .68 (autonomy) and .82 (participation).

### 3.3 Statistical analyses

IBM SPSS Statistics version 25 was used for all the statistical analyses. There were no missing values in the data set, as each question had to be responded to before moving on to the next question in the survey.

Pearson's correlations were used to assess the relationship between the study variables and hierarchical multiple regression analyses were used to examine how the explained variance of patient safety culture and work engagement was distributed among the predictors. Two multiple regression analyses were conducted with patient safety culture and work engagement as outcomes. Transformational leadership, job demands (work pace, mental workload, emotional workload), job resources (skill utilization, autonomy, participation) were the independent variables in both analyses, as well as work engagement in the analysis of patient safety culture. Gender and years of employment were adjusted for in both analyses. We conducted preliminary analyses of linearity, normality, multicollinearity and homoscedasticity, showing that none of the assumptions for conducting multiple regression analyses were violated.

## 4 RESULTS

The sample consisted of 139 home healthcare services employees, most of whom were female (96.4%). The distribution of age was relatively even among the age categories: 20–29 years (10.1%), 30–39 years (23.7%), 40–49 years (28.8%), 50–59 years (27.3%), 60+ years (10.1%). Most of the sample were healthcare personnel with high school education or a minimum of 3 years of higher education (Table 2). Years of employment ranged from less than 1–21 years or more (Table 2).

**Table 2:** Descriptive sample information (*N* = 139)

Occupational status	*N* (%)	Years of employment	*N* (%)
Managerial position	9 (6.5)	<1	6 (4.3)
Health personnel with min. 3 years higher education	59 (42.4)	1–5	38 (27.3)
Health personnel with high school education	56 (40.3)	6–10	35 (25.2)
Healthcare assistant (unskilled)	12 (8.6)	11–15	19 (13.7)
Administrative personnel	1 (0.7)	16–20	29 (20.9)
Other	2 (1.4)	=>21	12 (8.6)

The means, standard deviations and inter‐correlations for study variables are summarized in Table 3. As hypothesized, patient safety culture had a significantly positive correlation with work engagement, transformational leadership and the job resource dimensions and a negative correlation with the job demand dimensions. Work engagement had a significantly positive correlation with transformational leadership and the three job resources dimensions and a negative correlation with the job demand dimension “emotional workload.”

**Table 3:** Means, standard deviations (*SD*) and Pearson correlations between independent and dependent variables

Variables	Mean	*SD*	1	2	3	4	5	6	7	8	9
1. Patient safety culture	3.77	0.39	1								
2. Transformational leadership	3.47	0.81	.59**	1							
3. Work pace	2.57	0.63	−.42**	−.18*	1						
4. Mental workload	2.95	0.61	−.25**	−.11	.64**	1					
5. Emotional workload	1.96	0.40	−.43**	−.37**	.40**	.47**	1				
6. Skill utilization	2.85	0.52	.36**	.50**	−.11	.15	−.20**	1			
7. Autonomy	2.42	0.45	.29**	.31**	−.28**	−.11	−.27**	.49**	1		
8. Participation	2.54	0.55	.49**	.50**	−.30**	−.08	−.24**	.48**	.53**	1	
9. Engagement	5.48	1.1	.39**	.40**	−.14	−.05	−.18*	.45**	.29**	.29**	1

* *p* < .05.

** *p* < .001.

### 4.1 Patient safety culture

In the multiple regression analysis of patient safety culture, with gender, years of employment, transformational leadership, job demands (work pace, mental workload, emotional workload), job resources (skill utilization, autonomy, participation) and work engagement, transformational leadership was, as hypothesized, the strongest predictor (*β* = .30 *p* < .001) (Table 4). The model containing only the transformational leadership variable explained 35.7% of the variance in patient safety culture, while the full model, including all predictors explained 53.5% of the variance. In addition to transformational leadership, participation (*β* = .22 *p* < .05) and work engagement (*β* = .19 *p* < .05) had a significantly positive impact on patient safety culture; work pace (*β* = −.28 *p* < .05) had a negative impact.

**Table 4:** Hierarchical regression analysis for variables predicting patient safety culture (*N* = 139)

	Patient safety culture				
	Model 1	Model 2	Model 3	Model 4	Model 5
Variables	*β*	*β*	*β*	*β*	*β*
Gender	−.018	.028	−.028	−.036	−.025
Years employed	−.257*	−.110	−.121	−.127	−.160
Transformational leadership		.561**	.439**	.353**	.303**
Work pace			−.335**	−.285*	−.280*
Emotional workload			−.185*	.086	.105
Mental workload			.115	−.188*	−.197
Skill utilization				.005	−.066
Autonomy				−.066	−.079
Participation				.215*	.223*
Work engagement					.191*
*R* ^2^	.066	.357	.482	.509	.535
*F* for change in *R* ^2^	4.81*	61.21**	10.58**	2.33	7.180*

* *p* < .05.

** *p* < .001.

### 4.2 Work engagement

In the multiple regression analysis of work engagement, with gender, years of employment, transformational leadership, job demands (work pace, mental workload, emotional workload) and job resources (skill utilization, autonomy, participation), skill utilization was the strongest predictor (*β* = .37 *p* < .001) (Table 5). The predictive value of transformational leadership decreased substantially when the job resources dimensions were included in the model, but remained a significant positive predictor in the final model (*β* = .26 *p* < .05). None of the other predictor variables had a significant impact on work engagement. The model containing only the transformational leadership variable explained 17.5% of the variance in work engagement, while the full model, including all predictors explained 28.2% of the variance.

**Table 5:** Hierarchical regression analysis for variables predicting work engagement (*N* = 139)

	Work engagement			
	Model 1	Model 2	Model 3	Model 4
Variables	*β*	*β*	*β*	*β*
Gender	−.045	−.009	−.028	−.057
Years employed	.009	.123	.119	−.174
Transformational leadership		.432**	.411**	.259*
Work pace			−.115	−.026
Emotional workload			.060	−.102
Mental workload			−.015	.051
Skill utilization				.372**
Autonomy				.067
Participation				−.043
*R* ^2^	.002	.175	.183	.282
*F* for change in *R* ^2^	0.144	28.33**	0.435	5.89*

* *p* < .05.

** *p* < .001.

## 5 DISCUSSION

The central purpose of this study was to examine the associations between patient safety culture, transformational leadership, job demands and resources and work engagement. The results supported the first hypothesis, as transformational leadership, job resources and work engagement were all positively related to patient safety culture and job demands was negatively related to it. The second hypothesis was also supported, showing that transformational leadership was a stronger predictor for patient safety culture than job demands, job resources and work engagement. The strong association found in this study between transformational leadership and patient safety culture are consistent with previous research (Clarke, 2013; McFadden et al., 2009; Sfantou et al., 2017). Moreover, the results support the emphasis in international and national policies and guidelines on the importance of leadership and culture in quality and patient safety work. It is argued that leadership is the key determinant in developing and maintaining an organizational culture (Francis, 2013; Ministry of Health & Care Services, 2016; West, Eckert, Steward, & Pasmore, 2014). In some countries, such as Norway, the essential role and responsibility of managers in leading quality and safety improvement is strengthened in the regulations of the healthcare system (Ministry of Health & Care Services, 2012, 2016; NOU, 2015). Our study is the first to explore this association in Norwegian home care services, implying a stronger evidence base for policymakers' current emphasis on the role of managers and leadership in developing sound systems and culture for patient safety. In response to these findings, we argue that proper training and the further education of healthcare managers should include both theoretical and practical insight into these areas, as this is often not part of their training.

This study stands out, as it adds to previous research by exploring job demands and resources, as well as engagement and how these factors affect patient safety culture when adjusting for the role of leadership. Work pace (job demand), participation (job resource) and work engagement were also significant predictors of a patient safety culture. The negative relationship between job demands and patient safety culture is in line with other research findings (Phipps, Malley, & Ashcroft, 2012; Ramanujam, Abrahamson, & Anderson, 2008). Phipps et al. (2012) suggest, however, that if staff members are highly motivated and have adequate resources and support, their job demands might actually facilitate safety improvement. Managers play a key role in balancing job demands with job resources (Schaufeli, 2015), which in turn are related to patient safety culture.

This study partially supports the third hypothesis, in that transformational leadership and job resources were positively related to work engagement and the work demand dimension “emotional workload” was negatively related to work engagement. However, the work demand dimensions “work pace” and “mental workload” did not correlate significantly with work engagement. The last hypothesis in this study, that transformational leadership is a stronger predictor for work engagement than job demands and job resources, must be rejected, as the job resource “skill utilization” turned out to be the strongest single predictor for work engagement. Thus, employees' perceptions that their work is varied, providing them with opportunities for personal growth and development, to learn new things and to achieve something meaningful, is important for employees' work engagement.

A recent study of 675 Belgian home care nurses found that job resources were related to greater work engagement and less burnout (Vander Elst et al., 2016). Similarly, a meta‐analytic study by Nahrgang et al.. (2011) showed that job resources were related to engagement and safety outcomes. Their study looked at other types of resources, such as knowledge and a supportive and motivating environment and explored the associations across industries (Nahrgang et al., 2011). Although our study shows that job resources are more important for work engagement than transformational leadership, managers might have an indirect effect on work engagement through their impact on job demands and job resources, as suggested in a study by Schaufeli (2015). Our study suggests that managers' effort in facilitating a proper balance between job demands and resources, as well as increasing employees' work engagement by giving them opportunities for utilizing their skills and competencies, might be possible paths through which leaders might improve the patient safety culture.

### 5.1 Strengths and limitations

To our best knowledge, with the exception of one previous study (Olsen & Bjerkan, 2017), this is the first Norwegian survey‐based study of patient safety culture in home care services and the first to explore the associations between leadership, job demands and resources, work engagement and patient safety culture in this setting. The sample diversity due to location (rural/urban) and municipality and unit size reduces the likelihood of localization‐ and group‐specific effects.

We had a theoretical and empirical rationale for the postulated relationships between the study variables. However, the use of a cross‐sectional design is a limitation when drawing inferences about causal relationships, because all variables are assessed at the same point in time (Kestenbaum, 2009). Thus, future studies should explore the relationship between the study variables using a longitudinal design. Furthermore, as sub‐group analyses could not be justified in this study due to the small sample size, future studies should explore whether these relationships differ across occupations. For example, Wagner et al. (2018) found that hospital physicians and nurses differed in their perceptions of patient safety culture, with physicians rating the culture more positively than nurses did.

Caution should be used when generalizing the results, as the sample was small and selected purposively based on the units' participation in an intervention project (Wiig et al., 2018). However, it is difficult to recruit participants in this setting and the validity is strengthened by the high response rate of 67.5%. For comparison, the other Norwegian study on patient safety culture in home care had a response rate of 28% (Olsen & Bjerkan, 2017). The great predominance of women in the sample is representative for the home care setting in general (Helsedirektoratet, 2017), but caution should be used when generalizing to men and to other healthcare sectors.

## 6 CONCLUSIONS AND IMPLICATIONS

This study found that transformational leadership behaviours has a significant impact on patient safety culture and work engagement in home care services. Job demands and resources and work engagement also affect patient safety culture. The findings of this study highlight the importance of managers' use of transformational leadership in increasing work engagement among employees and improving patient safety culture in home care services.

From an organizational perspective, we recommend education and proper training of home care managers to increase their awareness of leadership styles, patient safety culture and to strengthen a transformational leadership style. Such interventions should also stress the managers' role in facilitating employees' possibilities for personal growth and development, participation in decision‐making processes and ensuring a proper balance between job demands and resources. From an organizational perspective, there is a need to provide managers with sufficient time and space to reflect on their responsibility as role models and leaders in patient safety. There is a need for collective reflexive spaces and arenas for home care managers to discuss patient safety. Our research has demonstrated how home care managers can take advantage of facilitated arenas and tools to support discussions of their role in developing a culture of patient safety (Johannessen et al., 2019). From a policy perspective, we recommend policy guidelines and regulations to enable making such efforts in healthcare organizations, by emphasizing the importance of transformational leadership for sound work environments and patient safety cultures and by providing resources to leadership and work environment interventions. We recommend national leadership programs targeted to managers in the home care context, as this differs significantly from the specialized healthcare context, where most effort has been invested in patient safety programs (The Norwegian Directorate of Health, 2019). Programs should focus on knowledge of the patient safety perspectives, methods and tools to instil home care managers with both the knowledge and the skills to improve the patient safety culture. Future research should further explore the relationship between the study variables using a longitudinal design.

## CONFLICT OF INTEREST

The authors declare that they have no competing interests.

## AUTHORS' CONTRIBUTIONS

ER was responsible for designing the study, data collection and administration of the survey, statistical analysis in SPSS, the interpretation of data and writing of the first draft of the manuscript. SW was responsible for the application for funding, recruitment, designing the study, data collection and commenting on drafts of the manuscript. Both authors have approved the final version.

## ETHICAL APPROVAL

The Regional Committees for Research Ethics in Norway found that the study was not regulated by the Health Research Act. The Norwegian Social Science Data Services approved the study (NSD, ID 52324). The study followed the Helsinki Declaration, and all participants gave their informed consent.

## Data Availability

Anonymized data sets of the study is available on request from the corresponding author.
